# Supplementary material for: Effectiveness of Social Measures against COVID-19 Outbreaks in Selected Japanese Regions Analyzed by System Dynamic Modeling
Source: Int J Environ Res Public Health. 2020 Aug 27;17(17):6238. doi: 10.3390/ijerph17176238 (PMC7503244; doi:10.3390/ijerph17176238)
Supplement: Supplementary file 1 [file ijerph-17-06238-s001.pdf]

International Journal of
Environmental Research and Public Health

Table S1. Representative data of confirmed positives by polymerase chain reaction (PCR) virus testing on and after 11 March 2020.

Days after 11 March 2020	Tokyo	Osaka	Hokkaido
0	6	7	7
5	23	35	41
10	69	52	48
15	192	83	57
20	454	171	66
25	965	335	83
30	1636	623	128
35	2376	895	191
40	3115	1223	340
45	3768	1403	490
50	4084	1553	656
55	4643	1615	780
60	4801	1666	843
65	4893	1688	888
70	4932	1697	908
75	-	-	946
80	-	-	973
85	-	-	993
90	-	-	1020

-: Data was not collected for analysis because disease spreading slowed down. The figures may differ from official announcement as official data can be corrected after verification.
